# Complete remission of multiple liver metastases with only partial response of the primary rectal cancer after neoadjuvant chemotherapy

**DOI:** 10.1186/s40792-020-00807-y

**Published:** 2020-02-27

**Authors:** Satomi Miura, Kyoji Ito, Nobuyuki Takemura, Fuminori Mihara, Tomomichi Kiyomatsu, Norihiro Kokudo

**Affiliations:** 1 Hepato-Biliary-Pancreatic Surgery Division, Department of Surgery, National Center for Global Health and Medicine, 1-21-1 Toyama, Shinjuku-ku, Tokyo, 162-8655 Japan; 2 Colorectal Surgery Division, Department of Surgery, National Center for Global Health and Medicine, 1-21-1 Toyama, Shinjuku-ku, Tokyo, 162-8655 Japan

**Keywords:** Colorectal liver metastases, Neoadjuvant chemotherapy, Liver-first strategy

## Abstract

**Background:**

Colorectal cancer is commonly diagnosed among the Japanese population, and various strategies in treating the colorectal liver metastasis have been introduced over the years. Here, we present a case of colorectal liver metastases in which we devised a multidisciplinary treatment plan for a better prognosis.

**Case presentation:**

We report a case of a 44-year-old female who developed rectal cancer with advanced synchronous liver metastases and was treated by a liver-first surgical approach following neoadjuvant chemotherapy. At diagnosis, there were 12 bilobular lesions in the liver, and the primary rectal cancer was asymptomatic and unprogressive. We adopted a liver-first strategy because the control of the liver metastases was considered the key prognostic factor. Furthermore, because the lesions were highly progressive, we planned neoadjuvant systemic chemotherapy first to provide an observational period to identify potential new metastatic lesions that were refractory to systemic chemotherapy or contraindicative for surgical resection. We administered two courses of S-1 + oxaliplatin (SOX)+ bevacizumab (BV) and an additional course of SOX without BV as neoadjuvant chemotherapy in preparation for surgery. This resulted in a prominent minimalization of colorectal liver metastases, and no other remote metastasis was observed. Then, surgical resection of the colorectal liver metastases was performed safely, and the pathological result revealed complete remission of all tumors by neoadjuvant chemotherapy. The primary tumor in the colon was successfully resected 2 months after the hepatectomy. Although the patient experienced a recurrence in two different sites in the lungs 10 months after resection of the primary rectal lesion, these metastases were successfully resected after diagnosis. The patient is alive with no signs of recurrence 3 years after the diagnosis of colorectal cancer with synchronous liver metastases.

**Conclusions:**

The combination of a liver-first strategy and neoadjuvant chemotherapy is a possible treatment of choice to cure colorectal cancer with simultaneous advanced colorectal liver metastases.

## Background

Globally, colorectal cancer (CRC) is the third most commonly diagnosed cancer among men and second among women. This is mirrored in the Japanese population [1]. The prognosis of CRC has improved over the recent years; stage IV colon cancer with distant metastasis is no longer considered incurable [2]. In particular, liver metastasis is one of the most common metastatic sites of colorectal cancer, and to date, surgical and chemotherapeutic approaches have been shown to improve the overall survival of the patients with colorectal liver metastases (CRLM) [2]. However, the treatment strategy for colorectal cancer with synchronous liver metastases remains to be an important issue. The order in which surgery should be performed (liver-first, colorectal-first, or simultaneous) and the efficacy of neoadjuvant chemotherapy are provoking controversy around the world [3].

In this article, we presented a case of colorectal cancer with advanced synchronous liver metastases which were treated by a combination of neoadjuvant chemotherapy and a liver-first surgical approach.

## Case report

**Fig. 1:**
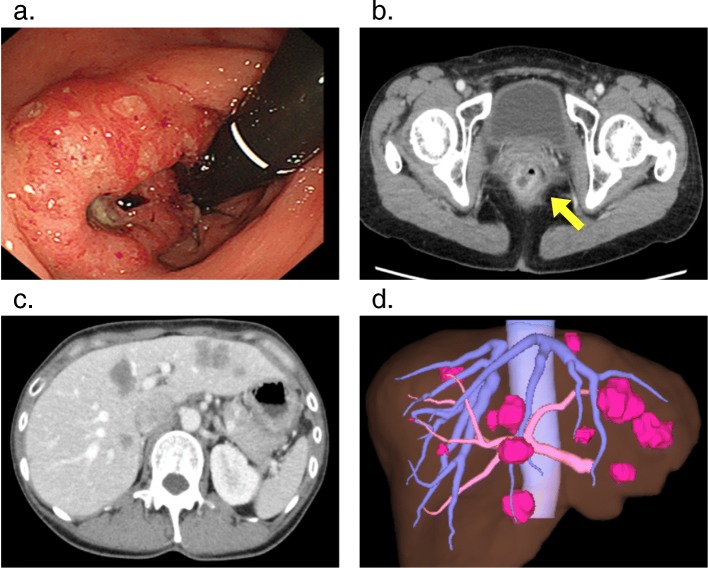
CT imaging and a colonoscopy before neoadjuvant chemotherapy. **a** Primary rectal cancer on a colonoscopy. Type 2 rectal carcinoma. No bleeding or occlusion is observed. **b A**xial view of primary rectal cancer (arrow) on CT. Contrast-enhanced wall-thickening (20 mm) is seen in the lower rectum. There were no signs of occlusion. **c** Axial view of multiple bilobular liver metastases on CT. **d** Multiple liver metastases on 3-dimentional image from CT. Lesions are bilobular. CT, computed tomography

**Fig. 2:**
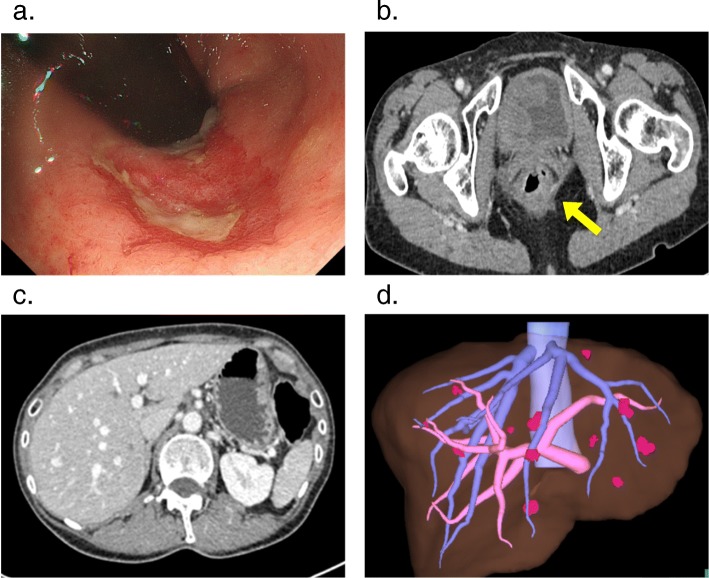
CT imaging and a colonoscopy after neoadjuvant chemotherapy. **a** Primary rectal cancer on a colonoscopy. The rectal cancer became flat and exceedingly shrunken. **b** Axial view of the primary CRC (arrow) on a CT. Contrast-enhanced wall-thickening (13mm) has been improved. **c** Axial view of multiple bilobular liver metastases on CT. The metastatic lesions shrunk prominently. **d** The 3-dimensional image of the multiple bilobular liver metastases. All metastatic lesions shrunk prominently. The estimated remnant liver volume was 73.4%. CRC, colorectal cancer; CT, computed tomography

**Fig. 3:**
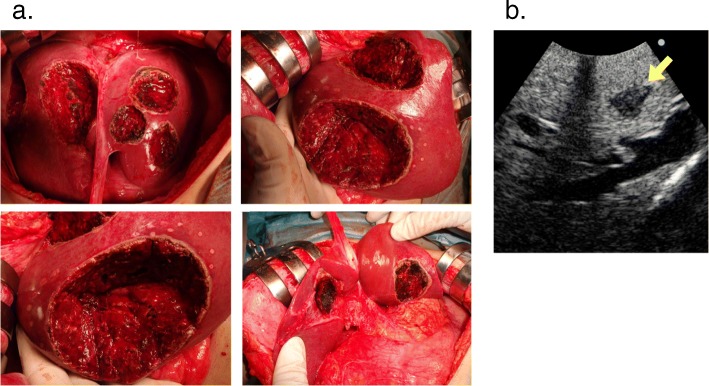
Intraoperative images in CRLM resection. **a** Resection surface of the liver after a hepatectomy. **b** Contrast-enhanced ultrasonography using Sonazoid. Shrunken CRLM lesions were visualized as defects. CRLM, colorectal liver metastases

**Fig. 4:**
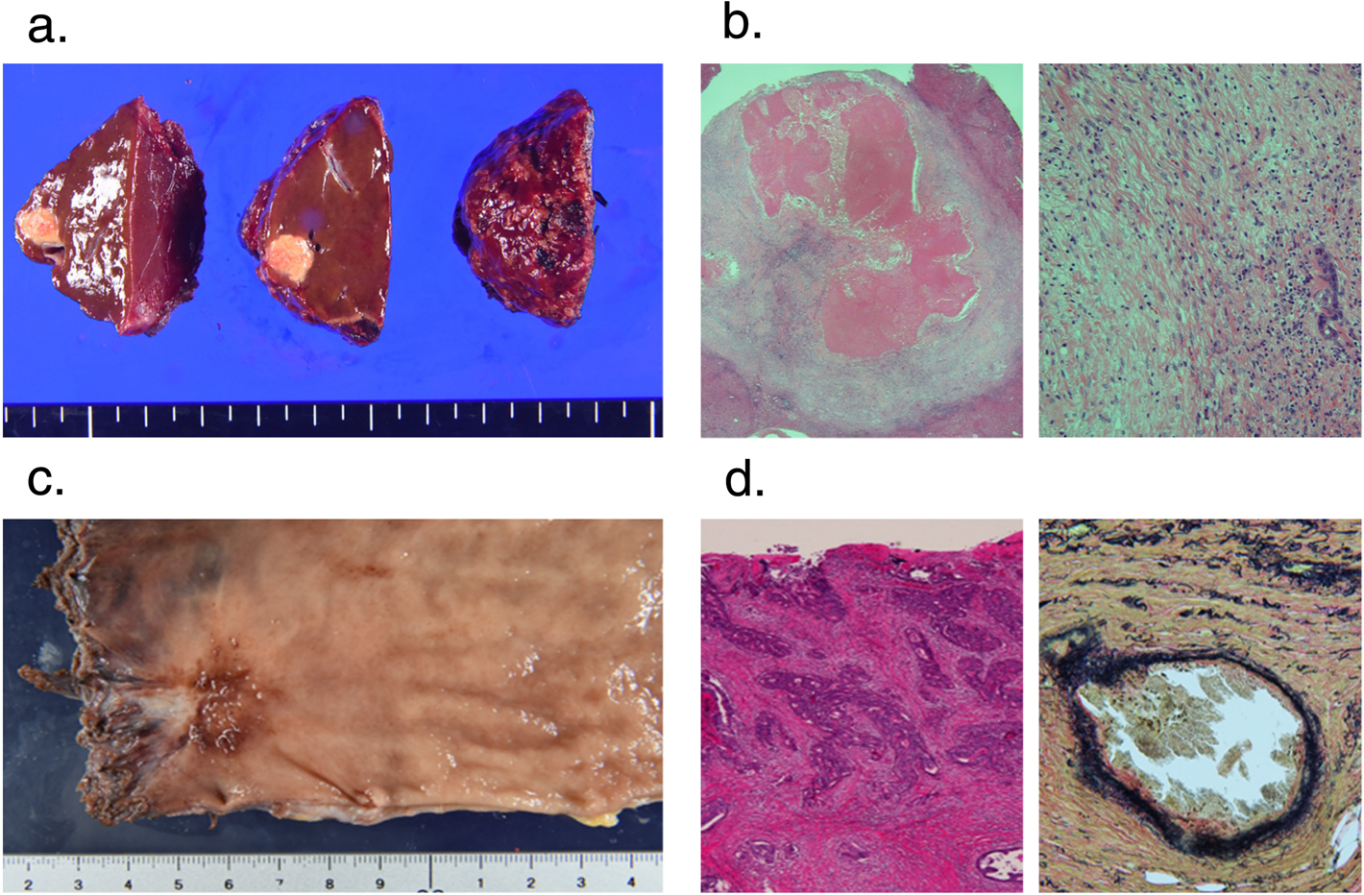
Pathological findings of the specimen. **a** Macroscopic view of the liver metastases lesion (S4). **b** Distant and short-range view of hematoxylin and eosin staining of the liver metastases lesion. No viable carcinomic cells were identified. **c** Macroscopic view of CRC lesion. **d** Distant view of hematoxylin and eosin staining (left) and short-range view of Elastica van Gieson staining (right) of the CRC lesion. Glandular structure of intermediate-differential adenocarcinoma and viable carcinomic cells were packed within a small vein. CRC, colorectal cancer

**Fig. 5:**
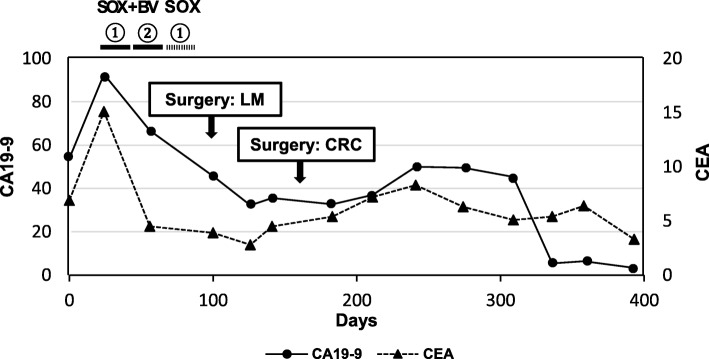
The clinical course of the patient and transition of tumor marker levels. CA 19-9 and CEA levels decreased after initiation of systemic neoadjuvant chemotherapy. CA 19-9, carbohydrate antigen 19-9; CEA, carcinoembryonic antigen

A 44-year-old woman was identified as having positive fecal occult blood and a halo within the left side of the liver upon ultrasonography at a regular medical check-up and was referred to our hospital in November 2017. A colonoscopy revealed a type 2 tumor in the rectum (Fig. 1a), and an adenocarcinoma was detected by biopsy from the tumor. Contrast-enhanced computed tomography (CT) showed a well-defined solid mass in the lower rectum (Fig. 1b) and bilateral and multiple (12 in total) low-attenuation lesions in the liver (Fig. 1c, d). The patient was diagnosed with rectal cancer with synchronous advanced liver metastases. Additional remote metastases were not detected, and therefore, surgical resection of the primary rectal cancer and liver metastasis was indicated. Due to the fact that the primary tumor in the rectum was small and the rectum was unobstructed, treatment for the CRLM was considered to be the key prognostic factor, and therefore, the liver-first strategy was adopted. At that stage, the indocyanine green retention rate after 15 min (ICG-R15) was 11.4%, and the estimated remnant liver volume was 61.1%; therefore, a liver surgery was considerable. However, because the CRLM lesions were highly progressive, we planned a neoadjuvant systemic chemotherapy first to provide an observational period to identify potential new metastatic lesions, which were refractory to systemic chemotherapy or contraindicative for surgical resection. Two courses of S-1 + oxaliplatin (SOX) + bevacizumab (BV) were administered (21-day cycle; day 1: oxaliplatin (130 mg/m^2^) + BV (7.5 mg/kg); day 1–14: S-1 (70 mg/m^2^/day)), and an additional course of SOX without BV was performed in preparation for surgery. At this point, the patient presented low white blood cell count (grade 2), generalized weakness (grade 2), loss of appetite (grade 1), and constipation (grade 1). The enforcement of chemotherapy resulted in a considerable reduction in the size of the tumor in both the rectum (Figs. 1a, b and 2a, b) and the liver (Figs. 1c, d and 2c, d). The primary tumor wall thickening was reduced from 20 mm to 13 mm on CT, and upon colonoscopy, the tumor was seen as a 20 mm size type 2 lesion before chemotherapy (Fig. 1a), whereas the tumor was prominently reduced to nothing but a flat granulation scar after chemotherapy (Fig. 2a). In addition, the pathology upon colonoscopic biopsy detected nothing but inflammatory cells, and therefore we have concluded that both the primary and CRLM lesions responded well to chemotherapy. On the course of chemotherapy, there was no great change in the serum albumin, total bilirubin, or prothrombin time, and no ascites was observed. ICG-R15 was 12.0% even after chemotherapy. The estimated remnant liver volume was 73.4% at this point (Fig. 2d). Considering the fact that the CRLMs were multiple and bilobular, the liver-first strategy was estimated to be suitable for the case. Eleven weeks after the introduction of neoadjuvant chemotherapy and 3 weeks after the final chemotherapy (8 weeks after the final administration of the final BV), a total of 10 partial hepatectomies were performed to extirpate 12 lesions. The clamp crushing method and the Pringle maneuver was adopted within the procedure (Fig. 3a). Because all tumors were located close to the liver surface, all lesions were accessed easily without division of main branches of the portal vein or hepatic vein. We used a contrast-enhanced ultrasonography by Sonazoid to detect the CRLM lesions, which were reduced after chemotherapy (Fig. 3b). The operative time was 7 h 12 min, and the total blood loss was 1334 ml. The postoperative course was uneventful, and the patient was discharged from the hospital on postoperative day 9. The pathology of the surgical specimen revealed nothing but scars replacing what supposedly were lesions of liver metastasis. No residue of cancerous cells was observed within the scar; therefore, it was concluded that complete remission was achieved through chemotherapy (Fig. 4a, b). Pathology revealed no findings of edema, fibrosis, or thrombosis around the central hepatic vein and the sinusoids in the background liver, indicating the fact that there were no signs of sinusoidal obstruction syndrome. Two months after the hepatectomy, a laparoscopic intersphincteric resection of the primary rectal tumor was performed. The operative time was 6 h 7 min, and the blood loss was 86 ml. The pathology showed a moderately differentiated adenocarcinoma penetrating into the muscularis propria. Most cancerous cells were viable (Fig. 4c, d). There was no lymph node metastasis. After further analysis, although the primary lesion seemed exceedingly diminished after neoadjuvant chemotherapy upon colonoscopy, the preoperative chemotherapy was defined as grade 1a, and we concluded that its effect on the primary focus remained limited. The patient had a recurrence in two different sites in the lung (a single node in the lower-right lobe and another in the lower-left lobe) 10 months after the resection of the primary CRC lesion. A 2-stage surgery was performed to resect the metastatic lesions in the lungs. Three years after the diagnosis of colorectal cancer with synchronous liver metastases, there have been no signs of recurrence and the patient is still alive. The clinical time course of the patient and the change in the levels of tumor markers are summarized in Fig. 5.

## Discussions

In the recent years, the prognosis of CRC has been improving [2]. Several treatment strategies are potentially key for improving the prognosis of patients with CRC. These include closer follow-up after resection and earlier detection of metastatic disease, improvement in chemotherapeutic regimens, and the enhancement of meetings held by multidisciplinary teams and tumor boards [4]. Typical metastatic lesions of colorectal cancer include the liver, lung, peritoneum, nodes, and ovary, with the liver being the most common site of metastasis overall [5].

In CRLM treatment, hepatic resection remains to be the only curative option. If macroscopic surgery is determined to be feasible and if the maintenance of at least 30% of future liver remnant may be sustained under R0 resection, surgery should be the first-line therapy [2]. If the patient has no known underlying illnesses, no prognostic factor will overturn the choice of treatment [6]. However, there is no standard approach with regard to the order of surgical resection for synchronous liver metastases of CRC. There are no differences reported among the colorectal-first (classic), liver-first (reverse), and simultaneous approaches [3]. Generally, if the patient is presenting with symptoms such as bleeding, obstruction, and/or perforation, CRC resection should be performed first followed by systemic chemotherapy and the resection of CRLM [7]. If the CRC lesion is asymptomatic, a liver-first strategy may be an option to prevent chemotherapy-induced liver damage before hepatectomy and progression of the CRLM lesions [8]. If the colorectal lesion is uncomplicated and the number of liver metastases is limited, simultaneous resection of all lesions may be chosen [9]. With any approach, it is important to consider the treatment plan independently for each case based on the characteristics of the CRC and CRLM through close observation of imaging modalities [10]. In the present case, the rectal lesion was asymptomatic and unprogressive, and the liver lesion was highly progressive. Therefore, CRLM was clearly considered to be a primary prognostic factor, and we adopted a liver-first strategy for the present case.

Although hepatic resection remains the only curative option for CRLM, long-term survival or curing the condition is reported to be realized in only 20–50% of patients who have undergone R0 resection of CRLM [11]. The meta-analysis reported by Kansas GP et al. demonstrated that approximately half of the patients with resectable liver-limited CRC metastases developed widespread systemic disease within 3 years of resection [12]. Although systemic chemotherapy is an alternative treatment option for CRLM, there is no single consensus for the peri-operative chemotherapy to CRLM, and the best criteria for perioperative chemotherapy is yet to be decided. In the ESMO guideline, published in 2016, for patients with unresectable CRLM who may withstand intensive therapy, an upfront active combination regimen is recommended [2]. Generally, if the patient has a good performance status with five or more CRLMs, portal node involvement, or bilobular CRLM lesions are noted, neoadjuvant chemotherapy is recommended before surgical resection. Neoadjuvant chemotherapy is effective in many ways; it helps to see whether surgical treatment is possible or not in terms of the identification of potential new metastatic lesions which were contraindicated for surgery and also prevents systemic progression of the tumor [13]. However, it is also reported that downstaging is only attained in 10–15% of patients who undergo neoadjuvant chemotherapy [14]. In the present case, the patient was young and presented with a good performance status at diagnosis, and the liver lesion was highly advanced presenting bilobular spread and 12 tumor lesions. Therefore, the patient was considered to be a suitable case for neoadjuvant chemotherapy to secure an observational period to confirm that there was no other distant metastasis, except CRLM.

Chemotherapy must be planned accounting for the patient’s liver function. Chemotherapy for over 16 weeks before surgical resection increases the risk of liver injury and post-operative complications, without pathologic improvement [15]. Long-term chemotherapy may lead to liver toxicity which may appear as symptoms, such as steatohepatitis [16] and portal hypertension [17]. In our case, the patient had a satisfactory liver function, and no obvious liver damage was seen along the course of chemotherapy.

Adam et al. reported an analysis of CRLM patients who underwent surgery in their institution, concluding that complete remission may occur in almost one-third of objective responders 60 years or younger with metastases ≤ 3 cm and with low carcinoembryonic antigen (CEA) values [18]. Our case met all of these conditions, which was a clear indication for the conduction of neoadjuvant chemotherapy, which resulted in a prominent minimalization of the CRLMs upon imaging. Surgical resection was then performed safely, and the pathological result revealed complete remission of all CRLMs by neoadjuvant chemotherapy. The primary tumor in the colon was successfully resected 2 months after CRLM resection. Therefore, neoadjuvant chemotherapy was clearly effective for this patient, and the liver-first strategy plus neoadjuvant chemotherapy could be a treatment choice to cure CRC with simultaneous advanced CRLM.

In our case, both the primary and the metastatic sites seemed to have a great sensitivity to neoadjuvant chemotherapy upon CT and colonoscopy. However, final pathological findings of the primary lesion revealed viable cells existing adjacent to the muscularis propria with vascular invasion, while the pathology for the liver lesions presented complete remission. Gervaz et al. compared histological response in colorectal liver metastases and the primary lesion in 29 patients, in which all were treated with a neoadjuvant chemotherapy followed by liver-first surgery, and then the primary lesion surgery [19]. There was a significant difference in the complete absence or significantly poor tumor response to chemotherapy between primary tumors (35.7%) and liver metastases (6.9%). Gervaz et al. hypothesized that a better drug delivery to the liver and the therapeutic interval of the primary lesion after CRLM resection causing repopulation of cancer cells may be a cause of worse tumor response in the primary lesion compared to the CRLMs. In our case, the surgery for the primary tumor was performed approximately 2 months after the resection of CRLMs, which could also affect the difference in the effectiveness of the neoadjuvant chemotherapy between the primary lesion and CRLMs.

In conclusion, neoadjuvant chemotherapy and a liver-first surgical approach could be a possible curable treatment for asymptomatic colon cancer with simultaneous advanced CRLM, especially for young patients with no rise in CEA and with no CRLM lesions over 3 cm in size. Neoadjuvant chemotherapy played a key role in treating unresectable, but potentially curable, metastatic CRC.

## Data Availability

The datasets analyzed during the current study are not publicly available due to their containing information that could compromise the privacy of research participants but are available from the corresponding author on reasonable request.

## Abbreviations

BV: Bevacizumab
CEA: Carcinoembryonic antigen.
CRC: Colorectal cancer
CRLM: Colorectal liver metastases
CT: Computed tomography
ICG-R15: Indocyanine green retention rate after 15 min
SOX: S-1 + oxaliplatin
